# Hypochlorous Acid as a Potential Wound Care Agent: Part I. Stabilized Hypochlorous Acid: A Component of the Inorganic Armamentarium of Innate Immunity

**Published:** 2007-04-11

**Authors:** L Wang, M Bassiri, R Najafi, K Najafi, J Yang, B Khosrovi, W Hwong, E Barati, B Belisle, C Celeri, MC Robson

**Affiliations:** a NovaBay Pharmaceuticals, Inc, Emeryville, CA; b Eye Institute San Rafael, CA; c Institute for Tissue Regeneration, Repair and Rehabilitation, Bay Pines, FL

## Abstract

**Objective:** Hypochlorous acid (HOCl), a major inorganic bactericidal compound of innate immunity, is effective against a broad range of microorganisms. Owing to its chemical nature, HOCl has never been used as a pharmaceutical drug for treating infection. In this article, we describe the chemical production, stabilization, and biological activity of a pharmaceutically useful formulation of HOCl. **Methods:** Stabilized HOCl is in the form of a physiologically balanced solution in 0.9% saline at a pH range of 3.5 to 4.0. Chlorine species distribution in solution is a function of pH. In aqueous solution, HOCl is the predominant species at the pH range of 3 to 6. At pH values less than 3.5, the solution exists as a mixture of chlorine in aqueous phase, chlorine gas, trichloride (Cl_3_^−^), and HOCl. At pH greater than 5.5, sodium hypochlorite (NaOCl) starts to form and becomes the predominant species in the alkaline pH. To maintain HOCl solution in a stable form, maximize its antimicrobial activities, and minimize undesirable side products, the pH must be maintained at 3.5 to 5. **Results:** Using this stabilized form of HOCl, the potent antimicrobial activities of HOCl are demonstrated against a wide range of microorganisms. The in vitro cytotoxicity profile in L929 cells and the in vivo safety profile of HOCl in various animal models are described. **Conclusion:** On the basis of the antimicrobial activity and the lack of animal toxicity, it is predicted that stabilized HOCl has potential pharmaceutical applications in the control of soft tissue infection.

A remarkable feature of the immune system is its ability to launch an effective response against invading pathogens by deploying a group of highly reactive chemicals, including oxidized halogens, oxidizing radicals, and singlet oxygen.^1^,^2^

As depicted in Figure 1, the precursor of these reactive oxygen species (ROS) is the oxygen radical (O_2_), which is generated by specialized immune cells—neutrophils, eosinophils, mononuclear phagocytes, and B lymphocytes.^1^–^9^ Production of ROS in these cells is accompanied by a significant rise in oxygen consumption, a series of events collectively referred to as the oxidative burst. The primary enzyme responsible for ROS production is a mitochondrial-membrane–bound enzyme known as respiratory burst NADPH oxidase.^1^ Patients with chronic granulomatous disease have oxidase defective genes, which makes them susceptible to repeated infection.^10^,^11^ During a respiratory burst, neutrophils produce H_2_O_2_, which is converted to HOCl by the activity of the granule enzyme myeloperoxidase in the following reaction.^12^


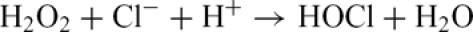


HOCl is known to be the major strong oxidant produced by neutrophils, and is a potent microbicidal agent within these cells.^2^,^10^ Experimentally, it has been estimated that 10^6^ neutrophils stimulated in vitro can produce 0.1 μM HOCl. This quantity of HOCl can kill 1.5 × 10^7^ *Escherichia coli* in less than 5 minutes.^13^ HOCl reacts readily with a range of biological molecules, particularly those with thiol, thiolether, heme proteins, and amino groups,^12^ and may lead to tissue injury. Taurine, a nonessential amino acid naturally found at roughly 15 mM within neutrophils acts as a scavenger molecule for HOCl via the following mechanism, and effectively dampens the collateral damage to cellular macromolecules caused by HOCl.^14^


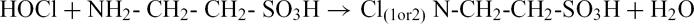


To date, pure HOCl has not been developed as a commercial pharmaceutical formulation presumably because of the challenge of maintaining storage stability. In this article, we describe a method for the preparation and stabilization of a pure form of HOCl (also referred to as NVC-101) for potential use as a pharmaceutical agent. We show here that when compared to the commercially available disinfectants hydrogen peroxide and sodium hypochlorite (NaOCl), this formulation has improved in vitro antimicrobial activity and therapeutic index. Furthermore, we present data demonstrating an excellent safety profile for NVC-101 in animal toxicology studies. We believe the improved properties of our pure physiologically balanced stabilized form of HOCl may allow for its use in a clinical situation such as in the treatment or prevention of infection in burn or other wounds.

## MATERIALS AND METHODS

### Preparation of HOCl

Reagent-grade NaOCl was purchased from J. T. Baker. Hypochlorous acid was prepared in 154 mM NaCl by acidifying reagent-grade NaOCl to the pH range of 3.5 to 4.0 with dilute HCl. A Beckman pH meter was used to accurately measure the final pH values. The concentration of active total chlorine species in solution expressed as [HOCl]_T_ (where [HOCl]_T_ = [HOCl] + [Cl_2_] + [Cl_3_^−^] + [OCl^−^]) in 0.9% saline was determined by converting all the active chlorine species to OCl^−^ with 0.1 M NaOH and measuring the concentration of OCl^−^. The concentration of OCl^−^ was determined spectrophotometrically at 292 nm (ε = 362 M^− 1^ cm^− 1^)^15^ with an Agilent 8453 UV-visible spectrophotometer.

### Microbiological materials

All microorganisms used in these studies were purchased from the American Type Culture Collection (ATCC), grown and propagated according to the recommendations for each strain by ATCC. Bacterial cells were harvested at stationary phase and concentrations were determined by 10-fold dilution as direct colony count. To prepare inoculum, bacteria were diluted in sterile saline before use to minimize the effect of broth on HOCl.

### Minimum bactericidal concentration

A modification of the National Committee Consensus on Laboratory standardized protocol “Methods for Dilution Antimicrobial Susceptibility Tests for Bacteria That Grow Aerobically” was used in these studies. Sterile 0.9% saline at pH 3.5 to 4.0 (vehicle) was used as the diluent. Use of such a diluent allows for the determination of the intrinsic activity of HOCl in the absence of any interfering molecules. Specifically, each test article is diluted using 2-fold serial dilution in acid-washed glass tubes to give a range of concentrations from approximately 2 to 0.002 mM in a final volume of 1 mL. Each dilution is inoculated with 5 × 10^5^ CFU/mL test bacteria and coincubations were carried out at room temperature for 60 minutes. At 60 minutes posttreatment, 0.1 mL of each reaction was immediately transferred into prelabeled 1.5-mL microfuge tubes containing 0.9 mL Dey and Engley (D/E) neutralizer broth (Hardy Diagnostic, Santa Maria, CA). Minimum bactericidal concentration (MBC) is determined by plating 0.1 mL of each sample onto an agar plate. Plates were then incubated overnight at 35°C, and examined for colony growth. The concentration at which there was a complete absence of colony growth is determined to be the MBC. Comparative MBC results provide estimates of the susceptibility of various test articles against test organisms.

### Time kill

For time kill studies, 5 mL of test article at an approximate MBC concentration was inoculated with approximately 10^6^ CFU/mL of each test organism and incubated for 0, 5, 10, 15, 20, 30, 60, and 90 minutes at room temperature. For each time point, 0.1 mL was transferred into 0.9 mL of D/E neutralizer broth and 0.1 mL of this mixture was plated and incubated as previously described.

### Cytotoxicity

L929 (ATCC CCL-1, NCTC clone 929) is a connective-tissue cell line derived from normal subcutaneous areolar and adipose tissue of a 100-day-old male C3H mouse. L929 cells were purchased from ATCC and propagated according to supplier's recommendations. These cells were then seeded at 1.5 × 10^4^ cells per well in 96-well plates and incubated overnight at 37°C. On the day of testing, growth medium was aspirated from each well, and 30 μL fresh medium was added per well. Test articles were diluted by 2-fold serial dilution using 154 mM saline at the desired pH for each test article. Following that, 170 μL of each dilution was added to each well for a total volume of 200 μL per well. After 60 minutes' exposure at 37°C, test articles were replaced with 200 μL of fresh tissue culture media and incubated for 24 hours at 37°C. Cell viability was determined by addition of WST-8 (Dojindo, Japan) reagent and the absorption at 450 nm read spectrophotometrically. Orange-red formazan, which is produced by live cells, is a direct measure of cell viability in this assay.

### Therapeutic index

The therapeutic Index of an antimicrobial agent is defined as the ratio of the concentration to achieve 50% cell toxicity (CT_50_) to MBC.

### Animal safety and toxicity studies

Ocular irritation, skin sensitization, and wound toxicology studies were performed. A preliminary (non–good laboratory practice [non-GLP]) study with a development formulation of HOCl (2.5 mM; 0.013% w/v) was carried out at the Brookdale Eye Clinic (K. Najafi, MD, unpublished data). Dutch pigmented rabbits received either 5% ophthalmic povidone-iodine (Betadine) (15 eyes) or the development formulation (15 eyes). Each eye received 0.1 mL of solution every 8 hours for a total of 72 hours and observations were made periodically during this time. The effect of the development formulation was compared to 5% ophthalmic-grade Betadine.

A GLP ocular irritation study (NAMSA, Toledo, OH) was designed to determine the potential for ocular irritation following a single instillation in the rabbit. New Zealand White rabbits (5 per group) were used. Hypochlorous acid (NVC-101) was instilled in the right eye at concentrations of 0.01%, 0.03%, and 0.1% w/v (pH 3.5). The left eyes were used as the controls and were untreated, vehicle (saline) or positive control treated. In all cases, the volume used was 0.1 mL, which was placed into the lower conjunctival sac. Evaluations for irritation were made at 24, 48, and 72 hours. At 24 hours, the cornea was examined using fluorescein stain.

GLP repeat-dose wound toxicity studies (Charles River, Spencerville, OH) were designed to provide maximum exposure to full-thickness wounds in rats and mini-pigs. Wounds were treated with NVC-101 at concentrations of 0.01%, 0.03%, and 0.1% w/v (pH 3.5). The test material was applied to the wounded area directly using soaked gauze. The treated site was covered for approximately 24 hours per day for 28 days. Wounds achieving 75% closure were kept open by abrasion. Parameters used to assess systemic toxicity included clinical signs (including observations of the site), body weights, food intake, clinical chemistries (blood and urine), hematology, organ weights, and gross and microscopic tissue evaluations.

## RESULTS

### Synthesis of hypochlorous acid

Hypochlorous acid can be synthesized by one of the 3 methods: hydrolysis of chlorine gas (eq 1), electrolysis of salt solution (eqs 2a and 2b), and acidification of hypochlorite (eq 3).


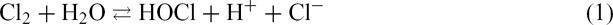



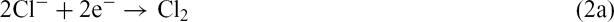



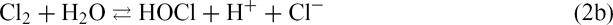



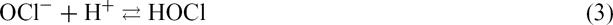


The limitations of using equation 1 are the inherent hazards of handling chlorine and the difficulty in manipulation. The disadvantage of the electrolysis method (eq 2) is the difficulty in controlling the target concentration of solution. Since hypochlorite is commercially available, the use of the method in equation 3 is the preferred method and is more convenient, safe, and controllable when compared to the other 2 methods.

### Distribution of active chlorine species

**Figure 2:**
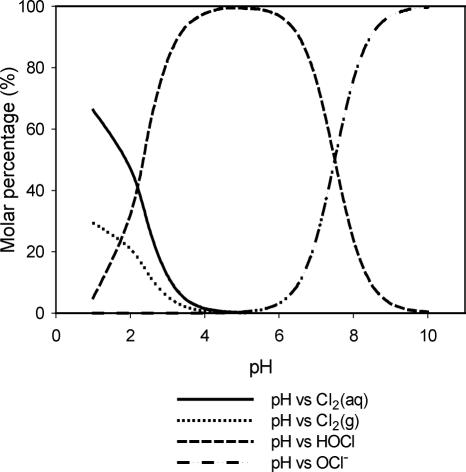
Chlorine speciation profile as a function of pH.

This section discusses the distribution of active chlorine species as a function of pH in physiologically balanced saline solution. The presence of Cl^−^ in HOCl solution could result in the formation of Cl_2_ and Cl_3_^−^. The formation of Cl_2_ has a significant impact on the stability of HOCl (Fig 2).

Equations 4 to 7 show the equilibria existing in HOCl/NaC1 solution.


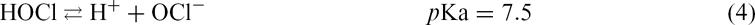



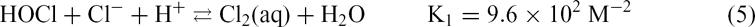



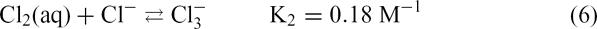



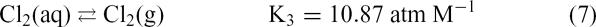


The molar percentage of each species in physiologically balanced HOCl solution is a function of pH (Fig 2). As is shown in equation 5, low pH and high [Cl^−^] favors the formation of Cl_2_. Once Cl_2_ is formed in the aqueous phase, it migrates into the headspace to reach the equilibrium shown in equation 7. The transfer of Cl_2_ from the solution to the headspace of the container results in a decrease in active chlorine concentration in solution. Therefore, the degassing of Cl_2_ becomes a major path for loss of HOCl in an open system (nonsealed). This is a potential problem for clinical use of HOCl. To stabilize the physiologically balanced HOCl solution, minimizing the formation of Cl_2_ is essential. Figure 2 shows the chlorine species distribution as a function of pH in accordance with the equilibria shown in equations 4 to 7. The lines are the calculated values based on the equilibrium constants shown in equations 4 to 7.^16^–^19^

### Microbicidal effect of HOCl

Stabilized HOCl demonstrates broad-spectrum antimicrobial activity at concentrations ranging from 0.1 to 2.8 μg/mL (Table 1).

The exception is *Aspergillus niger*, where a higher concentartion of HOCl (86.6 μg/mL) was required for effective killing of the organism under the same assay conditions. The overall summary of MBC findings against various microorganisms is shown in Table 1. Similarly, differences in HOCl sensitivity among different bacteria have been reported previously.^20^–^22^

### Time kill

Time kill is an in vitro measure of how fast a given antimicrobial can kill test bacteria. The rate of kill by stabilized HOCl was first demonstrated at the MBC values for each microorganism using an inoculum size of 1 × 10^6^ mL^−1^ for each test bacteria (Tables 2 and 3). As it is shown in this table, majority of test organisms were killed (>99.99%) within the first 2 minutes of exposure. Among the bacterial species tested, *Streptococcus pyogenes* 49399 was the only exception, which required approximately 10 minutes of exposure for effective killing, under the same assay conditions. The killing rate of stabilized HOCl with NaOCl and H_2_O_2_ was then determined against 3 specific test organisms—*E. coli 25922, P. aeruginosa* 27853, and *S. aureus* 29213—at room temperature for a total of 90 miniutes.

It is worth mentioning that all these time kill studies were also performed with an inoculum size of 1 × 10^7^/mL for each test bacteria, and the comparative results are depicted in Table 4.

**Figure 3:**
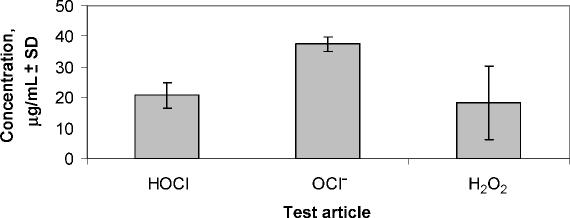
Comparative time kill studies of HOCl, NaOCl, and H_2_O_2_ against 3 test organisms—*Escherichia coli* 25922, *Pseudomonas aeruginosa* 27853, and *Staphylococcus aureus* 29213—at room temperature for total of 90 miniutes.

As the results show, HOCl at its MBC values for different test organisms (5.6–12.5 μM) was able to kill all 3 test bacteria in less than 1 minute, with no significant bacterial killing effect from its excipient, saline at pH 4.0 (data not shown). However, the kill time for NaOCl at MBC values 10 to 50 μM varied from 5 to 15 minutes for the same 3 test organisms. In contrast, H_2_O_2_was only able to kill *P. aeruginosa* 27853 at 7500 μM in about 10 minutes, but did not kill *S. aureus* 29213 at its highest concentration tested (20,000 μM) even up to 90 minutes' exposure time under the same assay conditions (Fig 3).

### Comparative cell toxicity

**Figure 4:**
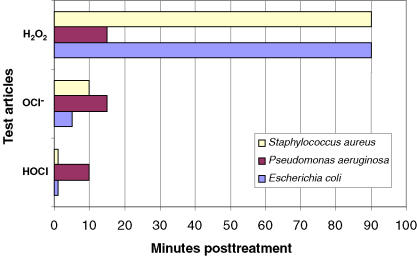
Relative cell toxicity of hypochlorous acid (HOCl; pH 4.0), hypochlorite (OCl^−^; pH 10.5), and hydrogen peroxide (H_2_O_2_; pH 7.0) on L929 cells. Cytotoxicity measured in a cell proliferation assay is expressed as the concentration (μg/mL) that reduces the cell number by 50% of vehicle-treated control. CT_50_ is shown as the average of 7, 3, and 5 independent experiments (consisting of 10 different concentrations of each test article plus/minus the standard deviation), respectively.

The relative cell toxicity of HOCl, NaOCl, and H_2_O_2_ was assessed following a standard method used to examine the cytotoxicity of liquid disinfectants.^23^ This toxicity assay utilizes an established adherent cell line, L929, and the end point is relative cell viability measured by addition of WST-8 (Dojindo, Japan) colorimetric reagent. Orange-red formazan, which is produced by live cells, is a direct measure of cell viability in this assay. Cytotoxicity was measured using 2-fold dilutions of HOCl, NaOCl, and H_2_O_2_ as compared to untreated or vehicle-treated control L929 cells. The CT_50_ was calculated for each test article, the values for which are shown in Figure 4. The CT_50_ values for HOCl (15–25 μg/mL) and NaOCl (38–42 μg/mL) were reproducible and closely matched published results for NaOCl.^16^ However, the CT_50_ values for H_2_O_2_ were more variable (5–35 μg/mL), probably due to the chemical instability of H_2_O_2_ under these assay conditions.

### Relative therapeutic index

**Figure 5:**
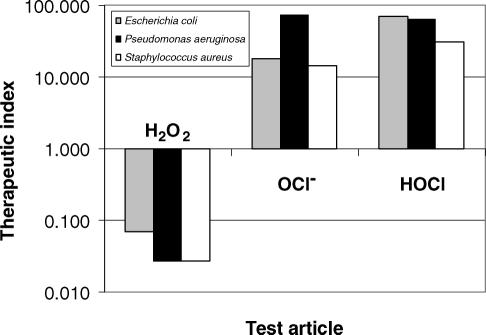
Relative therapeutic index of hypochlorous acid (HOCl; pH 4.0), hypochlorite (OCl^−^; pH 10.5), and hydrogen peroxide (H_2_O_2_; pH 7.0). Therapeutic index is expressed as a ratio of the CT_50_ concentration (μg/mL) on L929 cells divided by the minimum bactericidal concentration (μg/mL) for *Staphylococcus aureus* 29213, *Pseudomonas aeruginosa* 27853 and *Escherichia coli* 2592. The higher the therapeutic index, the safer the test article will be.

The therapeutic indices for HOCl, NaOCl, and H_2_O_2_ were assessed using L929 cells and 3 clinically relevant bacterial strains—*E. coli* 25922, *P. aeruginosa* 27853, and *S. aureus* 29213. The calculated therapeutic index values for all 3 organisms are summarized in Figure 5.

The value for stabilized HOCl is approximately 98-fold higher than that for H_2_O_2_ for the gram-negative bacterium *E. coli* 25922, and more than 1000-fold higher than H_2_O_2_ for gram-positive organisms like *S. aureus* 29213.

### Animal safety and toxicity

Stabilized HOCl is reactive, and therefore is not persistent. To evaluate its potential toxicity, several well-established animal models were used. Stabilized HOCl was found to be nonirritating in (rabbit eye) and nonsensitizing in (guinea pig) animal models (Table 5). No ocular irritation was observed following the instillation of a development formulation (0.013% HOCl) into the eyes of Dutch pigmented rabbits every 8 hours for 72 hours (data not shown). Stabilized HOCl at concentrations of 0.01%, 0.03%, and 0.10% w/v in a standard Buehler-design dermal sensitization study in guinea pigs showed no evidence of dermal reaction. Similarly, 28-day toxicity studies in full-thickness wounded rats and mini-pigs with daily application of stabilized HOCl at 0.01%, 0.03%, and 0.1% w/v together with a 24-hour occluded dressing showed no evidence of systemic toxicity. Microscopic examination of the wound area showed the expected signs of wounding and subsequent wound repair. A summary of all toxicological safety results with stabilized HOCl is shown in Table 5.

## DISCUSSION

The germicidal properties of HOCl have been well reported.^1^,^10^,^13^,^20^,^21^,^24^ Hypochlorous acid is widely used as a disinfectant, for example, in sanitizing wash solutions and swimming pools. In these applications, the reactive chemical is formed in solution by the addition of chlorine to water. Similarly, HOCl is used to treat drinking water and is formed following addition of chlorine gas or NaOCl.

Figure 2 shows the relative molar distribution of various chlorine species in a closed saline solution system as a function of pH. Between pH levels of 3 and 6, the predominant species is HOCl. At higher pH, hypochlorite ion (OCl^−^) is formed, whereas at lower pH, the solution exists as a mixture of chlorine (Cl_2_) in solution, chlorine gas in the headspace, and HOCl. The control of this reaction has been utilized in industrial practices to optimize the availability of the active antimicrobial, HOCl.

In this report, stabilized HOCl is prepared by the addition of NaOCl to a solution of sodium chloride in sterile water, followed by addition of a solution of hydrochloric acid to form the active component, HOCl. Stabilized HOCl (referred to as NVC-101) is a dilute solution of HOCl in 150 mM (0.9%) sodium chloride at an unbuffered pH of 3.5. The solution is stored in inert sealed containers designed for maximum product stability.

As shown in Figure 2, in a pH range of 3 to 6 the predominant species is HOCl. At pH values greater than 5.5, hypochlorite ion (OCl^−^) is formed, and around pH 7.5 (the *p*Ka of HOCl of the chlorine species in solution is at 50/50% mixture [HOCl/OCl^−^]).^12^ As the pH increases from 9.5, the concentration of OCl^−^ in solution reaches its maximum level, becoming 100% hypochlorite (also referred to as bleach). However, on the acidic side at pH less than 4, the solution exists as a mixture of chlorine (Cl_2_) in aqueous phase, chlorine gas in the headspace, trichloride (Cl_3_^−^), and HOCl. At pH less than 3, an appreciable amount of Cl_2_ gas forms, which may cause the rapid loss of all active chlorine in an open container. To keep the solution stable and maintain its desired activity, the pH of the solution should remain between 3.5 and 5 and the solution should be stored in a tightly sealed container. For the first time, we have been able to determine these conditions to stabilize HOCl and to assess its biological properties as a pharmaceutical product.

The biological effect of HOCl on bacteria has been extensively studied.^22^,^25^,^26^ HOCl has broad-spectrum antimicrobial activity and is able to kill microorganisms very rapidly. Respiratory loss in bacterial cell membrane as a result of an irreversible reaction of HOCl with sulfur- and heme-containing membrane enzymes and structural proteins^12^ lead to cell death and nonviability.^21^,^22^,^25^

Topical antiseptics with a long history of use, such as NaOCl (Dakins' solution), hydrogen peroxide, acetic acid, and povidone-iodine remain in widespread use today. These antimicrobial agents used at typical concentrations are cytotoxic and impede wound healing, and so are now discouraged by some experts for use on chronic ulcers. NVC-101 is a low-concentration, acidified, unbuffered solution of HOCl in saline. Under the conditions of the formulation, the active ingredient is primarily HOCl in equilibrium with a small amount of dissolved chlorine. The studies presented here have shown that stabilized HOCl exhibits rapid, concentration-dependent activity against a wide variety of gram-negative and gram-positive bacteria, yeast, and fungal pathogens, as long as the narrow effective pH range is maintained. In vivo, HOCl is produced intracellularly in abundance in response to phagocytosis of pathogens by neutrophils and plays an important role in the destruction of pathogens.

HOCl, the active ingredient of stabilized HOCl (NVC-101), has rapid and broad-spectrum antimicrobial activity against clinically relevant microorganisms in vitro and in vivo. Although vegetative bacteria are more susceptible to NVC-101 than endospore-forming bacteria and fungi (Table 1), NVC-101 is fully capable of inactivating all groups of gram-negative and gram-positive bacteria, yeast, and fungi, including *S. aureus*, methicillin-resistant *S. aureus*, vancomycin-resistant *E. faecium* (Table 1), and *Bacillis anthracis* spores (data now shown). NVC-101 has been shown to be nonirritating and nonsensitizing in animal models. There was no evidence of ocular irritation following a single instillation of NVC-101 in the eyes of New Zealand White rabbits at concentrations of 0.01%, 0.03%, and 0.1% w/v. No ocular irritation was observed following the instillation of a development formulation in the eyes of Dutch pigmented rabbits every 8 hours for 72 hours (data not shown). NVC-101 at concentrations of 0.01%, 0.03%, and 0.1% w/v in a standard Buehler-design dermal sensitization study in guinea pigs showed no evidence of dermal reaction.

The active ingredient is reactive, and therefore is not persistent. Its persistence of antimicrobial properties has not yet been tested in the in vivo wound environment. Thus, absorption and systemic toxicity are expected to be insignificant. Therefore, in the 28-day wound toxicity studies in rats and mini-pigs with daily application of NVC-101 at 0.01%, 0.03%, and 0.1% w/v, with 24-hour occluded dressing, there was no evidence of systemic toxicity. Furthermore, microscopic examination of the wound area showed the expected signs of wounding and subsequent wound repair.

Heggers and colleagues^26^ have investigated the toxic effects of various concentrations of NaOCl (at pH 7.5, this was actually a 50:50 mixture of NaOCl and HOCl) in vitro and in vivo in the rat incision model. Concentrations used in previous studies were often quite high and although they had antimicrobial properties, they also exhibited some local toxicity that was not desirable. Heggers et al. conducted their experiments in the range of concentrations they expected to be active but not toxic to the cells or detrimental to wound healing. The concentrations evaluated were 0.25%, 0.025%, and 0.0125% w/v in the in vitro studies and 0.25% and 0.025% w/v in the in vivo studies. Ten clinical isolates were used in the in vitro studies (both gram-positive and gram-negative species). The bactericidal potential of the 3 concentrations was determined. All concentrations killed gram-positive bacteria within 30 minutes, but the lowest concentration did not kill gram-negative bacteria. Mouse fibroblasts were exposed to various concentrations of NaOCl for 10-, 20-, or 30-minute intervals. These cells remained viable except at the highest concentration, where cell death by 10 minutes was noted. In the incision rat model, 3 (2.5-cm) full-thickness wounds were created on each animal. The incisions were closed and the covered gauze was saturated every 4 hours with the NaOCl or saline. Subsets of animals were sacrificed on days 3, 7, and 14. Tissue sections were collected. Breaking strength was measured (force required to rupture the scar, in kilograms). The values for the breaking strength were higher as a function of duration, but the treated and control groups were not different. This study concluded that the concentration of 0.025% retains its bactericidal property without causing injury to the fibroblast cells.

Dakins' solution (NaOCl) has been used as an antimicrobial for decades. A study to assess the bactericidal activity and toxicity of 0.5% and 0.1% NaOCl was undertaken.^27^ Only the toxicity portion of the study is discussed here. The insult to guinea pig skin was assessed following application of 0.5% solution of NaOCl buffered to a pH of 7.49 for up to 2 weeks (soaked gauze resoaked every 8 hours). The animals were sacrificed on day 1, 4, 7, or 14. The hair was removed from the skin before application but the skin was intact. The application of 0.5% solution resulted in basal cell toxicity (15% decrease in viability after 2 weeks of treatment), and so a lower concentration of 0.1% solution was evaluated (pH 7.4). This lower concentration did not result in toxicity to the basal cells. Control and treated skin sites were similar when the microscopic morphology was evaluated. Epidermal hyperplasia and an inflammatory influx were noted in the treated animals at 2 weeks. The authors concluded that the solutions were therapeutic candidates for thermal injury. It is important to note that at pH 7.4, these solutions will have approximately equimolar quantities of HOCl and NaOCl.

In the present comparative studies, we have demonstrated that H_2_O_2_ and hypochlorite (NaOCl) are effective against certain bacteria (more effective against gram negatives, but not gram positives). However, those effective antimicrobial concentration ranges begin to correlate with higher cytotoxicity on mammalian cells, as compared to NVC-101. This antiseptic profile of NaOCl resemble some of the over-the-counter antiseptics: silver nitrates or silver ions, Betadine, and acetic acid (data not shown). Moreover, there are other antiseptics that are less toxic to mammalian cells but at the same time have lower antimicrobial activity (ethanol, hydrogen peroxide, and 5% mafenide acetate solution) as compared to NVC-101 (data not shown). The Department of Health and Human Services discourages the use of commonly used antiseptic solutions to treat wound infection in general and chronic nonhealing wounds in particular because, for reasons mentioned above, their uses are contraindicated. Therefore data presented in this study should help in selecting safer antimicrobial agents for wound disinfection, irrigation, and dressing. This reevaluation of accumulated evidence is intended as a basis to help practitioners make informed decisions for choosing the appropriate topical antimicrobial for wound care management.

As the development of bacterial resistance to antibiotics continues and controversy regarding the use of topical antiseptics persists, the need for research and development of new classes of antimicrobial agents that are safe and broadly effective and have low toxicity and low propensity to induce antimicrobial resistance becomes inevitably critical. Currently, the use of broad-spectrum topical antibiotics to treat wounds that are failing to heal or those at risk for getting infected is not recommended by the Department of Health and Human Services.^28^ These recommendations are based on the following reasons: antibiotics may cause allergic reactions; especially when applied topically may have lower tissue distribution; greater effect on endogenous microflora (disturbance of the normal commensal microflora); induce resistance; and eventually have reduced therapeutic efficacy. By the same token, antiseptics are not encouraged because of their higher toxicity, potential development of resistance (like antibiotics), and, more important, direct impact on wound healing process.

Efficacy of topical antimicrobial agents in the management of serious infections, for example, biofilm- and catheter-related wounds, particularly when chronic and nonhealing, is inconclusive. These observations vary greatly because of (*a*) inconsistent in vitro test specifications, (*b*) use of different animal species models, (*c*) use of different organisms for determining the efficacy outcome. Therefore, overall results make direct comparisons less than ideal. While in vitro testing is required to select potential agents for clinical trials, these models will never totally mimic in vivo conditions.

Thus, in light of published results on HOCl and data obtained in the present investigations, there is enough compelling evidence to show that our new formulation of HOCl (NVC-101), which resembles the HOCl molecule made by neutrophils during oxidative burst (a natural defense process against invading microorganisms), could lend itself for safe and effective treatment modalities of infection. Because of NVC-101's broad-spectrum, fast-acting antimicrobial activity, the chances of developing resistance would be minimal and based on its safety profile the potential for collateral damage to infected tissues is also very low. Therefore, in vivo experiments in a chronic infected granulating wound model are planned to determine NVC-101's ability to persist in a hostile environment where the pH range may not be ideal and where inflammation may produce exudates that limit its use as a wound care agent.

## Figures and Tables

**Figure 1:**
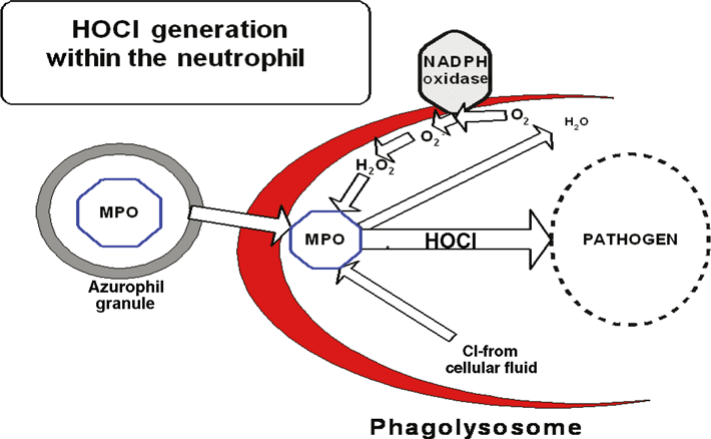
A schematic representation of hypochlorous acid (HOCl) production during the oxidative burst process. During this process, cells utilize O_2_ and convert it to hydrogen peroxide (H_2_O_2_) using a mitochondrial-membrane–bound enzyme NADPHase. Then, myeloperoxidase catalyzes the reaction between H_2_O_2_ and Cl^−^ to generate HOCl. As deregulations take place, the lumen of the phagasome progressively becomes more acidic and leaves the bacterium within a vacuole (phagolysosome) containing MPOse and H_2_O_2_ in a medium containing 0.1 M Cl^−^ at estimated pH 4 to 6. During this process, conditions are optimal for MPOse-catalyzed generation of HOCl as depicted in this figure. On the basis of these principles, we set out to establish the conditions of generating the stable form of HOCl (NVC-101).

**Table 1:** Minimum bactericidal concentration (μg/mL) of HOCl for a broad spectrum of microorganisms tested at room temperature for 60 min

**Pathogen**	**ATCC**	**MBC**
*Escherichia coli*	25922	0.7
*Pseudomonas aeruginosa*	27853	0.35
*Staphylococcus aureus*	29213	0.173
*Staphylococcus epidermidis*	12228	0.338
*Micrococcus luteus*	7468	2.77
*Corynebacterium amycolatum*	49368	0.169
*Haemophilus influenzae*	49144	0.338
*Proteus mirabilis*	14153	0.340
*Staphylococcus hominis*	27844	1.4
*Staphylococcus haemolyticus*	29970	0.338
*Staphylococcus saprophyticus*	35552	0.35
*Candida albicans*	10231	2.7
*Klebsiella pneumoniae*	10031	0.7
*Serratia marcescens*	14756	0.169
*Streptococcus pyogenes*	49399	0.169
*Enterobacter aerogenes*	51697	0.676
*Candida albicans*	10231	0.17
*Aspergillus niger*	16404	86.6
Methicillin-resistant *Staphylococcus aureus*	33591	0.682
Vancomycin-resistant *Enterococcus faecium*	51559	2.73

ATCC indicates American Type Culture Collection; MBC, minimum bactericidal concentration.

**Table 2:** Time kill for stabilized hypochlorous acid at MBC concentrations against different pathogens at room temperature

	**Time kill (min), recovered CFU/mL × 10^7^ (*n* = 3)**								
**Pathogen**	**0**	**1**	**2**	**5**	**10**	**15**	**30**	**60**	**90**
*Escherichia coli* 25922	0	0	0	0	0	0	0	0	0
*Pseudomonas aeruginosa* 27853	0	0	0	0	0	0	0	0	0
*Staphylococcus aureus* 29213	>60	0	0	0	0	0	0	0	0
*Staphylococcus epidermidis* 12228	0	0	0	0	0	0	0	0	0
*Micrococcus luteus* 7468	0	0	0	0	0	0	0	0	0
*Corynebacterium amycolatum* 49368	0	0	0	0	0	0	0	0	0
*Haemophilus influenzae* 49144	0	0	0	0	0	0	0	0	0
*Proteus mirabilis* 14153^a^	0	0	0	0	0	0	0	0	0
*Staphylococcus hominis* 27844	0	0	0	0	0	0	0	0	0
*Staphylococcus haemolyticus* 29970	0	0	0	0	0	0	0	0	0
*Staphylococcus saprophyticus* 35552	0	0	0	0	0	0	0	0	0
*Klebsiella pneumoniae* 10031	0	0	0	0	0	0	0	0	0
*Serratia marcescens* 14756	0	0	0	0	0	0	0	0	0
*Streptococcus pyogenes* 49399	>300	>300	>300	>300	>300	52	0	0	0
*Enterobacter aerogenes* 51697	0	0	0	0	0	0	0	0	0
*Candida albicans* 10231	0	0	0	0	0	0	0	0	0
*Asperigillus niger* 16404	>300	120	0	0	0	0	0	0	0
Methicillin-resistant *Staphylococcus aureus* 33591	>300	0	0	0	0	0	0	0	0
Vancomycin-resistant *Enterococcus faecium* 51559	>300	0	0	0	0	0	0	0	0
*Escherichia coli* 25922	0	0	0	0	0	0	0	0	0

**Table 3:** Comparative time kill studies of HOCl, NaOCl, and H_2_O_2_ against 3 test organisms at room temperature for a total of 90 min

		**Time kill (min)**		
**Pathogen**	**ATCC**	**HOCl**	**OCl^−^**	**H_2_O_2_**
*Escherichia coli*	25922	0	<5	>90
*Pseudomonas aeruginosa*	27853	<1	<20	<15
*Staphylococcus aureus*	29213	0	<10	>90

ATCC indicates American Type Culture Collection.

**Table 4:** Comparative MBC (μM) of HOCl, NaOCl, and H_2_O_2_ tested against 3 organisms at room temperature for 60 min

		**MBC (μM)**		
**Pathogen**	**ATCC**	**HOCl**	**OCl^−^**	**H_2_O_2_**
*Escherichia coli*	25922	5.6	40	7,500
*Pseudomonas aeruginosa*	27853	6.2	10	>20,000
*Staphylococcus aureus*	29213	12.5	50	>20,000

ATCC indicates American Type Culture Collection; MBC, minimum bactericidal concentration.

**Table 5:** Safety studies with control vs stabilized HOCl in 4 different animal species

**Studies**	**Species**	**Site applied**	**NVC-101 (% w/v)**	**Results**
Eye irritation	Rabbits	Eye, Q8 for 72 h	Saline	No irritation
			0.013	No irritation
			Betadine	Progressive irritation
Eye irritation	Rabbits	Eye, single instillation	Saline	No irritation
			0.01, 0.03, and 0.1	No irritation at any dose
Skin sensitization	Hartley-derived albino guinea pig	Skin	Saline	No sensitization
			0.01, 0.03, and 0.1	No irritation at any dose
28-Day toxicology	Rat	Full-thickness wound	Saline	No systemic toxicity at any dose and histopathology consistent with wound healing
			0.01, 0.03, and 0.1	
28-Day toxicology	Mini-pig	Full-thickness wound	Saline	No systemic toxicity at any dose and histopathology consistent with wound healing
			0.01, 0.03, and 0.1	
